# Adaptations to cationic biocide exposure differentially influence virulence factors and pathogenicity in *Pseudomonas aeruginosa*

**DOI:** 10.1080/21505594.2024.2397503

**Published:** 2024-09-16

**Authors:** Germán G. Vargas-Cuebas, Christian A. Sanchez, Elise L. Bezold, Gabrielle M. Walker, Shehreen Siddiqui, Kevin P.C. Minbiole, William M. Wuest

**Affiliations:** a Department of Microbiology and Immunology, Emory University School of Medicine, Atlanta, GA, USA; b Department of Chemistry, Emory University, Atlanta, GA, USA; c Department of Chemistry, Villanova University, Villanova, PA, USA

**Keywords:** Disinfectant, virulence factors, antimicrobial resistance, cross-resistance, *Pseudomonas aeruginosa*

## Abstract

Cationic biocides (CBs), which include quaternary ammonium compounds (QACs), are employed to mitigate the spread of infectious bacteria, but resistance to such surface disinfectants is rising. CB exposure can have profound phenotypic implications that extend beyond allowing microorganisms to persist on surfaces. *Pseudomonas aeruginosa* is a deadly bacterial pathogen that is intrinsically tolerant to a wide variety of antimicrobials and is commonly spread in healthcare settings. In this study, we pursued resistance selection assays to the QAC benzalkonium chloride and quaternary phosphonium compound P6P–10,10 to assess the phenotypic effects of CB exposure in *P. aeruginosa* PAO1 and four genetically diverse, drug-resistant clinical isolates. In particular, we sought to examine how CB exposure affects defensive strategies and the virulence-associated “offensive” strategies in *P. aeruginosa*. We demonstrated that development of resistance to BAC is associated with increased production of virulence-associated pigments and alginate as well as pellicle formation. In an *in vivo* infection model, CB-resistant PAO1 exhibited a decreased level of virulence compared to wild type, potentially due to an observed fitness cost in these strains. Taken together, these results illustrate the significant consequence CB resistance exerts on the virulence-associated phenotypes of *P. aeruginosa.*

## Introduction

Disinfectants stand at the front lines in the fight against bacterial infections, which are associated with one in eight deaths worldwide [1]. Effective infection prevention and control rests on the ability to eliminate human pathogens from high-contact surfaces and provide sterile surfaces in healthcare settings [2], though disinfectant use extends to a broader range of settings including agriculture, food industry, cosmetics, and domestic cleaning [3]. Chemical disinfectants fall into different classes including chlorine and chlorine-releasing compounds, peroxides, phenolics, and cationic biocides (CBs). CBs are among the most widely used disinfectants and include subclasses such as biguanides (e.g. chlorhexidine) and quaternary ammonium compounds (QACs) like benzalkonium chloride (BAC) and didecyl dimethyl ammonium chloride (DDAC) [4]. These cationic surfactants act upon bacteria by disturbing their cell envelopes, leading ultimately to membrane lysis and death [2].

However, in recent years, resistance to disinfectants such as CBs has emerged, being further exacerbated following the COVID-19 pandemic, which resulted in a significant increase in disinfectant usage thereby inducing pressure toward disinfectant resistance [5]. In addition to extended surface survival, disinfectant resistance can lead to profound phenotypic implications in bacteria that are equally concerning. For example, disinfectant resistance has been associated with an increase in virulence factor production, reduced metabolism and growth rate, and increased biofilm production [6,7].

**Figure 1.**
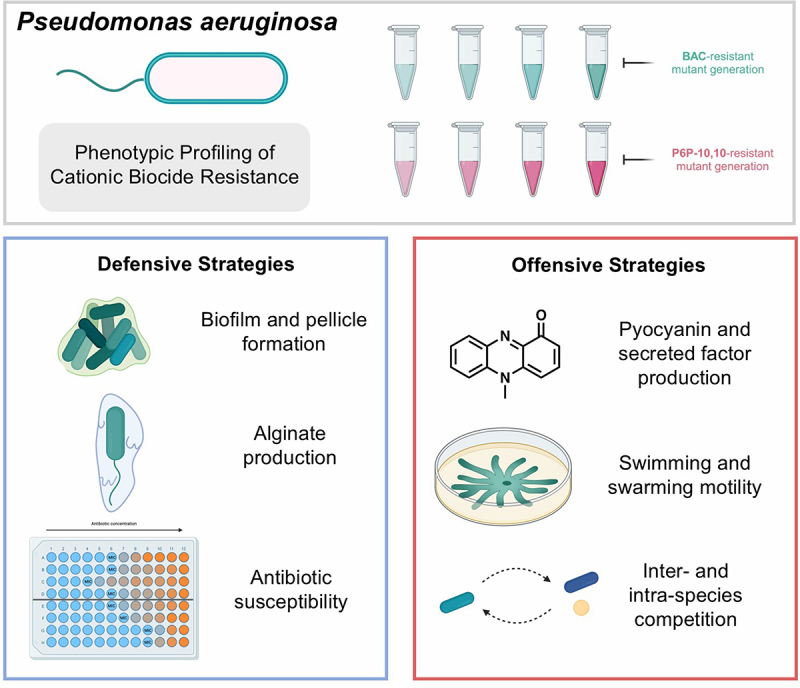
Schematic overview of *P. aeruginosa* virulence factors assessed after adaptation to cationic biocides (CBs). *P. aeruginosa* strains were exposed to increasing concentration of CBs over 15 d, and virulence-associated phenotypes were subsequently evaluated in these CB-resistant strains. These factors were grouped into “defensive” strategies that allow the bacterium to survive harsh conditions and external insults, and “offensive” strategies that allow the bacterium to attack, outcompete, or invade another organism. Created with BioRender.com.

*Pseudomonas aeruginosa* is an opportunistic gram-negative bacterial human pathogen of particular concern. Notoriously difficult to treat due to its wealth of resistance mechanisms to antibiotics and disinfectants alike, *P. aeruginosa* is responsible for over half a million deaths per year worldwide [8]. Through a combination of defense strategies and virulence factors, *P. aeruginosa* can establish deadly infections in immunocompromised hosts, especially in cystic fibrosis patients [9]. This bacterium possesses several defense strategies that protect it from the innate immune system and exogenous molecules like antibiotics. Biofilm and pellicle formation, alginate production, and intrinsic and acquired drug resistance mechanisms provide extensive protection for *P. aeruginosa* in infection settings [10]. Furthermore, the pathogenicity *P. aeruginosa* lies in what can be regarded as its “offense” strategies [11,12]. This includes secreted factors (pyocyanin, pyoverdine, and proteases), cellular motility, and additional ways of gaining competitive advantages in the presence of other organisms; these factors are summarized in Figure 1. Concerningly, *P. aeruginosa* can be transmitted from one person to another through contaminated equipment or surfaces in healthcare settings [13]. Because of its high priority rating by the CDC and the WHO, it is crucial to assess the effect that exposure to cationic biocides has on the defensive and offensive strategies of *P. aeruginosa*.

Prolonged subinhibitory exposure to disinfectants has become an environmental reality due to the increased CB prevalence in nature [14]. Previous reports have demonstrated that long-term exposure to BAC can promote antibiotic resistance in bacteria including *P. aeruginosa*. [6,15,16] Kim *et al*. demonstrated that isolates of *P. aeruginosa* from river sediment that were exposed to BAC over a 3-year time span developed cross-resistance to clinically relevant antibiotics such as ciprofloxacin and chloramphenicol. McCay *et al*. showed that after 33 serial passages of a *P. aeruginosa* clinical isolate in subinhibitory concentrations of BAC, the strain became less susceptible to ciprofloxacin; however, susceptibility to polymyxin B increased. Loughlin *et al*. also reported the development of serially passaged *P. aeruginosa* strains in sublethal amounts of BAC to explore antibiotic cross-resistance. In their studies, it was observed that the BAC-resistant mutants exhibited an increase in resistance to polymyxin B with no MIC changes against imipenem, ciprofloxacin, and tobramycin.

In this study, we explored the effects of sublethal CB exposure utilizing *P. aeruginosa* strains from a recently developed panel of diverse clinical isolates from the Multidrug-Resistant Organism Repository and Surveillance Network (MRSN) [17]. By using isolates from this panel, we are able to probe strains from a wide range of antibiotic susceptibility profiles and genetic backgrounds in addition to the laboratory reference strain PAO1. From the panel, we selected the pan-resistant isolate MRSN6220, extensively drug-resistant strains MRSN6241 and MRSN5524, and multidrug resistant strain MRSN409937. The disinfectants selected for this study included the most common CB BAC, used in a wide range of applications like hospital-surface disinfection, wound sterilization, pool water disinfection, and eggshell sanitization [3], as well as the novel quaternary phosphonium compound P6P–10,10, previously reported by our research groups [18]. This compound is a promising biocide, showing significantly higher potency (~100X) compared to other commercial disinfectants [18]. By comparing these two dramatically different cationic biocides, we hope to assess whether structural diversity in CBs influences the adaptation phenotypes in *P. aeruginosa* after long-term exposure.

Herein, we disclose that exposure to a pair of disparate CB structures results in changes in bacterial defensive and offensive phenotypes with distinct implications for pathogenesis. Exposure to structurally distinct biocides can lead to differential production of virulence factors and overall bacterial physiology of *P. aeruginosa* strains. This underlines the importance of the potential implications of disinfection protocols on virulence and pathogenicity, and calls for the rational selection and usage of disinfectants.

## Results and discussion

### CB resistance in P. aeruginosa influences antibiotic susceptibility

In our study, we isolated CB-resistant *P. aeruginosa* strains after exposure to subinhibitory concentrations of BAC and P6P–10,10 for 15 d. To evaluate the effect of CB resistance development on antibiotic susceptibility, we tested our CB-resistant and parental strains against 12 antibiotics with differing modes of action including aminoglycosides, monobactams, cephalosporins, carbapenems, fluoroquinolones, and polymyxins. No significant trends were observed in cross-resistance between antibiotics and CB resistance, in contrast with the previous reports mentioned. Nevertheless, our results suggest that some resistance development in CBs may increase susceptibility to certain antibiotic classes in *P. aeruginosa* (Table 1). The BAC-resistant strains of MRSN6241 and MRSN409937 showed enhanced susceptibility to aminoglycosides, while the BAC-resistant PAO1 strain showed reduced sensitivity to aminoglycosides. Previously, aminoglycoside antagonism has been observed in PAO1 resulting from a reduction in membrane polarization as a tolerance mechanism [19]. In two clinical isolates, however, an increase in susceptibility is observed. For the BAC-resistant MRSN6241 strain, a drastic change in aminoglycoside susceptibility shifts the MIC for gentamicin and tobramycin back into a clinically susceptible range compared to the parental strain. In addition to changes in aminoglycoside susceptibility, PAO1 and each MRSN strain had a CB-resistant derived strain that displayed an increased susceptibility to a fluoroquinolone antibiotic. Most notably, again, BAC-resistant MRSN6241 had a three-to-four-fold decrease in MIC for ciprofloxacin and levofloxacin. Additionally, we observed a two-to-three-fold decrease in MIC for the BAC-resistant mutant of MRSN409937 when tested against cephalosporins and carbapenems (Supplemental Figure S1). The “P6P^R^” strains showed little to no MIC difference in most cases, although the P6P–10,10-resistant mutant of MRSN409937 was markedly more susceptible to the three aminoglycosides tested. Overall, our results contrast with the previous studies which reported cross-resistance or no change for BAC-resistant *P. aeruginosa* clinical and environmental isolates.

**Table 1.** Minimum inhibitory concentrations (MICs) of parental (bold) and CB-resistant(R) strains of *P. aeruginosa*. Notable MIC differences are highlighted in red.

Cross Resistance MICs, ∞M					
Strain	Aminoglycosides			Fluoroquinolones	
	AMK	GEN	TOB	CIP	LVX
**PAO1**	4	2	0.5	2	8
PAO1 BAC^R^	8	8	2	1	4
PAO1 P6P^R^	4	4	1	4	8
**6220**	250	125	>250	125	250
6220 BAC^R^	250	125	>250	63	125
6220 P6P^R^	250	125	>250	125	125
**6241**	8	125	63	125	125
6241 BAC^R^	1	16	8	8	16
6241 P6P^R^	8	125	125	125	125
**409937**	16	8	4	16	63
409937 BAC^R^	4	2	1	8	63
409937 P6P^R^	1	0.25	0.25	16	63
**5524**	16	>250	250	4	32
5524 BAC^R^	16	>250	125	2	16
5524 P6P^R^	4	250	32	2	16

### CB resistance increases alginate production in P. aeruginosa

We sought to assess whether our resistance selection efforts resulted in any CB-resistant strains that increased alginate production to mitigate the effects of disinfectant treatment. The exopolysaccharide alginate encapsulates *P. aeruginosa* cells and has been shown to protect the bacteria from threats such as phagocytosis and disinfectant treatment [20–22]. *P. aeruginosa* strains infecting the lungs of cystic fibrosis (CF) patients frequently become overproducers of alginate, and this mucoid phenotype is crucial for establishing chronic infections in the lungs of CF patients [23]. It has been previously discussed that alginate production can be stimulated through various factors such as nitrogen limitation or high oxygen environments. To our knowledge, there have been no previous observations on whether CBs could influence alginate production in *P. aeruginosa*; though alginate has been shown to protect against QACs such as BAC and CTAB [22].

**Figure 2.**
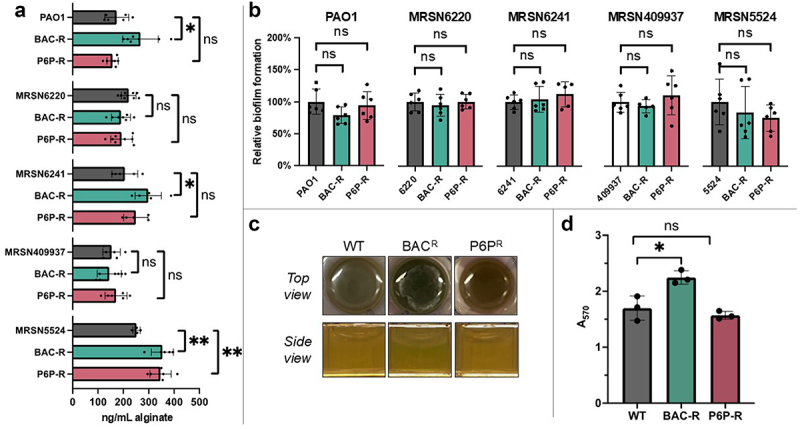
Evaluation of defensive strategies produced by *P. aeruginosa* CB-resistant strains. a) alginate production was quantified in a mixture of boric acid/sulphuric acid/carbazole using a spectrophotometer (A_550_) after alginate precipitation with 2% cetylpyridium and collection with isopropanol. The amounts of alginate produced were compared between the cb-resistant *P. aeruginosa* strains and their respective parental (WT) strain. b) relative biofilm formation by cb-resistant and parental strains was evaluated in M63 media and quantified using crystal violet measuring absorbance at a wavelength of 570 nm. c) pellicle formation of cb-resistant and parental PAO1 strains after overnight incubation. Differences in pellicle formation were clearly observed after overnight incubation with a thicker white film formed in the air–liquid interface (top and side view) in the bac-resistant strain compared to the P6P-resistant or PAO1 parental strains. d) pellicle formation was quantified using crystal violet and measuring absorbance at a wavelength of 570 nm (A_570_). The means and individual values for three biological replicates are shown. Statistical significance in pellicle formation between strains was determined using the two-tailed Student’s t-test comparing the A_570_ values. *, *p* < 0.05 and ns, not significant. Statistical significance in alginate and biofilm production between strains was determined using the two-tailed Student’s t-test. ns = not significant, ****p < 0.0001, ***p < 0.0005, **p < 0.005, *p < 0.05.

We quantified alginate production of both the parental and CB-resistant strains and found a significant increase in alginate production in the BAC-resistant strains of PAO1, MRSN6241, and MRSN5524, and in the P6P–10,10-resistant strains of MRSN5524. Our findings suggest that the QAC BAC is more likely to induce this phenotype change in *P. aeruginosa* compared to quaternary phosphonium compound P6P–10,10 (Figure 2a). Due to the rise of BAC resistance in the environment, this increased alginate phenotype could have profound implications for CF patients with CB-resistant *P. aeruginosa* infections.

### Quantification of biofilm in CB-resistant strains of P. aeruginosa

Bacteria are commonly found in bacterial aggregates called biofilms. Within these biofilms bacteria engage in community-like behaviors that enhance their survival including acquisition of nutrients, cooperative and competitive interactions, and protection from antimicrobials [24]. Biofilms are composed of a matrix of extracellular polymeric substances produced by bacteria that include nucleic acids, lipids, secreted proteins, polysaccharides, and water [25,26]. Biofilm development renders pathogens less susceptible to antimicrobials such as antibiotics and disinfectants, which can lead to recalcitrant infections. For example, Henly *et al*. reported that uropathogenic strains of *E. coli* adapted to BAC showed increased biofilm formation [27].

In *P. aeruginosa*, the main components of biofilms are the polysaccharides Pel (positively charged), Psl (charge-neutral), and alginate (negatively charged) [28]. Production of Pel and Psl is observed during infection of cystic fibrosis patients and reduces the effectiveness of antimicrobial treatments, while alginate is the main polysaccharide produced by mucoid strains and is associated with chronic infections [29,30]. Since we observed increased alginate production associated with CB resistance, we sought to first quantify biofilm production at the solid–liquid interface in the parent and CB-resistant strains using crystal violet staining in a minimal medium.

Surprisingly, no differences were observed in biofilm formation at the liquid–surface interface between the CB-resistant strains derived from the laboratory reference strain PAO1 or the strains derived from the clinical isolation panel (Figure 2b). When compared to the parental PAO1 strain, the BAC-resistant strain produced on average less biofilm compared to the other isogenic strains, however this reduction in biofilm production was not significant. A similar result was also observed in the BAC-resistant strain derived from MRSN409937 clinical isolate. Interestingly, the CB-resistant strains derived from MRSN5524 showed lower overall biofilm production, but not in a significant manner. Biofilm formation measured in lysogeny broth (LB) rich media also showed no significant difference between the CB-resistant strains and the parental strains (data not shown). Our data indicate that CB resistance does not lead to increased biofilm formation at the solid–liquid interface in *P. aeruginosa*.

### Pellicle formation is increased in the bac-resistant but not in the P6P-resistant strain derived from PAO1

Bacteria can also form floating biofilms at the air–liquid interface known as pellicles [31]. Pellicles are mainly composed of exopolysaccharides, usually cellulose. In *P. aeruginosa*, the pellicle matrix is composed of the Pel and Psl polysaccharides which are rich in glucose and in mannose, respectively [31]. Regulation of pellicle formation is complex and species-specific, and its matrix composition varies between bacterial species. Pellicle formation in the air–liquid interface allows bacteria to readily acquire oxygen from the air and nutrients from the media [31,32]. In *Bacillus subtilis*, exposure to sub-lethal concentrations of the biocide chlorine dioxide activates the membrane-bound histidine kinase KinC promoting biofilm formation in the form of pellicle [33]. Pellicle formation in *P. aeruginosa* requires proteins encoded in the *pel* and *psl* operons responsible for the synthesis of the required polysaccharides to build the matrix [34].

When the CB-resistant strains derived from PAO1 were grown in LB, we observed an evident increase in pellicle formation in the BAC-resistant compared to the other isogenic strains (Figure 2c). The perceived increase in biofilm biomass at the air–liquid interface suggested that pellicle formation was enhanced in the BAC-resistant strain. To quantify this difference in pellicle formation, we used biofilm peg plates and performed the assay as previously described [35]. We found a significant increase in pellicle formation in the BAC-resistant strain when compared to the parental isogenic strain (Figure 2d). No significant difference in pellicle formation was observed in the P6P-resistant strain. These data indicate that CB resistance differentially influences biofilm formation at the air–liquid interface, with only BAC resistance promoting pellicle formation in *P. aeruginosa*.

### BAC adaptation reduces swimming behavior in clinical isolates

Motility processes such as swimming and swarming play a vital role in pathogenesis of *P. aeruginosa* infections. Swimming – single cellular movement with flagella – aids *P. aeruginosa* in locating an infection site, whereas the swarming motion, which is coordinated with multicellular movement with flagella, is useful for the development of biofilms [36]. Together, these motility processes are multifaceted and assist in the pathogen’s defence to the host immune response. Previous studies have shown that exposure to a range of biocides can negatively affect motility in gram-negative species. Nordholt *et al*. observed a reduction in motility for *E. coli* BAC-resistant mutants [37]. This same result was observed in a long-term *E. coli* BAC exposure study, where the authors hypothesized that downregulation of motility could be a survival mechanism as the energy to produce flagella is high, thus this energy could be invested in other stress response strategies [38]. In a similar experiment where *E. coli* was serially passaged against BAC, Forbes *et al*. found that there was a reduced expression of genes related to motility [39]. Similar observations were found for gram-positive *Listeria monocytogenes* as BAC-adapted mutants exhibited reduced swarming motility [40].

Wanting to build upon this knowledge, we interrogated the relationship between CB resistance of *P. aeruginosa* clinical isolates and motility. As shown in Table 2, we observed that generally, the BAC-resistant strains exhibited less swimming behavior with the BAC-resistant mutant of MRSN6220 being markedly less motile. Although there were no apparent trends in swarming motility, it was observed that the BAC-resistant strain of MRSN6241 was less motile compared to the parent and P6P–10,10-resistant strains. These data suggest that BAC resistance can affect the swimming motility of *P. aeruginosa* and clinical isolates, which reflects the findings in previous studies of motility in gram-negative and -positive bacteria.

**Table 2.** Swimming and swarming behavior in parental (bold) and resistant (R) strains of *P. aeruginosa*. Diameters of swimming and swarming zones were calculated by averaging two perpendicular measurements of motility zones. Two biological replicates per strain were performed.

Strain	Swimming Diameter (mm)	Swarming Diameter (mm)
**PAO1**	37.0	7.5
PAO1 BAC^R^	33.0	6.5
PAO1 P6P^R^	36.5	5.0
**6220**	42.0	7.0
6220 BAC^R^	6.0	4.0
6220 P6P^R^	38.5	5.0
**6241**	35.0	21.5
6241 BAC^R^	28.0	8.5
6241 P6P^R^	30.0	18.0
**409937**	40.0	9.5
409937 BAC^R^	24.0	5.5
409937 P6P^R^	40.0	6.0
**5524**	12.0	9.5
5524 BAC^R^	6.5	5.0
5524 P6P^R^	8.5	5.0

### BAC resistance results in an increase in virulence-associated pigment production

**Figure 3.**
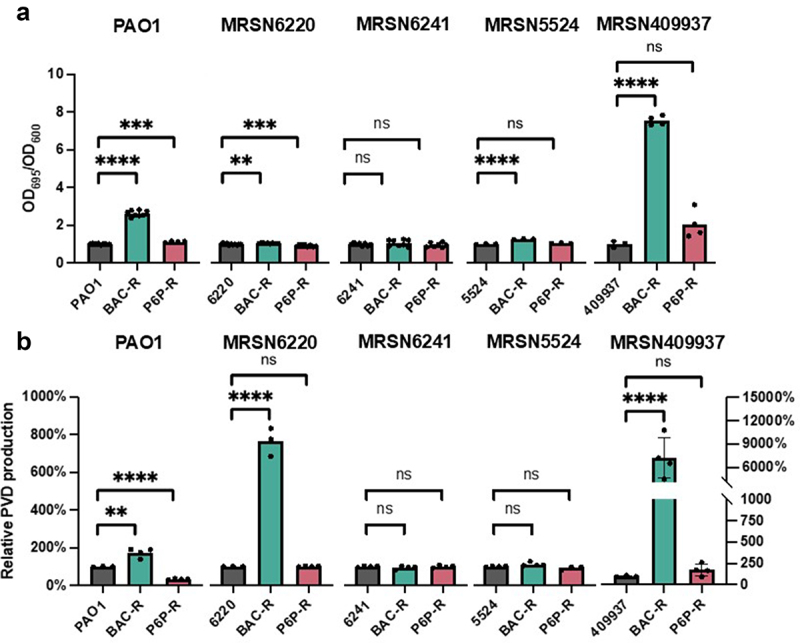
Evaluation of virulence-associated pigment production in *P. aeruginosa* cb-resistant strains. a) pyocyanin (PYO) production was measured spectrophotometrically from supernatants at a wavelength of 695 nm and normalized to the cell density using OD_600_ values. The normalized values from cb-resistant strains were compared to their respective parental strains. b) pyoverdine (PVD) production was assessed fluorometrically taking advantage of fluorescence of apo-pvd. PVD production of cb-resistant strains was compared to their respective parental strains. Statistical significance in PVD and PYO production between strains was determined using the two-tailed Student’s t-test. ns = not significant, ****p < 0.0001, ***p < 0.0005, **p < 0.005, *p < 0.05.

Due to their characteristic greenish-blue hue, pseudomonads are easily recognizable due to their production of vibrant pigments. In *P. aeruginosa*, these colorful compounds are primarily the siderophore pyoverdine (PVD) and redox active metabolite pyocyanin (PYO) [41,42]. Both compounds are associated with *P. aeruginosa* virulence and can be used to gain an advantage over human hosts and other bacteria in their environment. The relationship between pigment production and CB resistance has been mostly overlooked; although increased expression of pyoverdine biosynthesis genes has been observed previously in BAC-adapted *P. aeruginosa* strains without further phenotypic validation noted [43]. In contrast, it has been reported that a QAC-adapted strain of *P. aeruginosa* decreased in pyocyanin production [44]. We observed a sharp increase in PYO production compared to the parental strains in BAC-resistant strains derived from PAO1 and MRSN409937 (Figure 3a). In contrast, P6P–10,10 resistance appeared to have little impact on PYO production. In addition to PYO, the production of the fluorescent siderophore PVD was studied. Similar to PYO, sharp increases in PVD production were observed in BAC-resistant PAO1 and MRSN409937, and an increase was also observed in BAC-resistant MSRN6220 (Figure 3b). These phenotypic shifts in pigment production are visible when culturing in solid or liquid media. Because increased PVD and PYO synthesis are known to inhibit the growth of other bacteria, we hypothesized that BAC-resistant strains might have an increased competitive advantage against other bacteria.

### Lysis of S. aureus by disinfectant-resistant Pseudomonas aeruginosa PAO1

**Figure 4.**
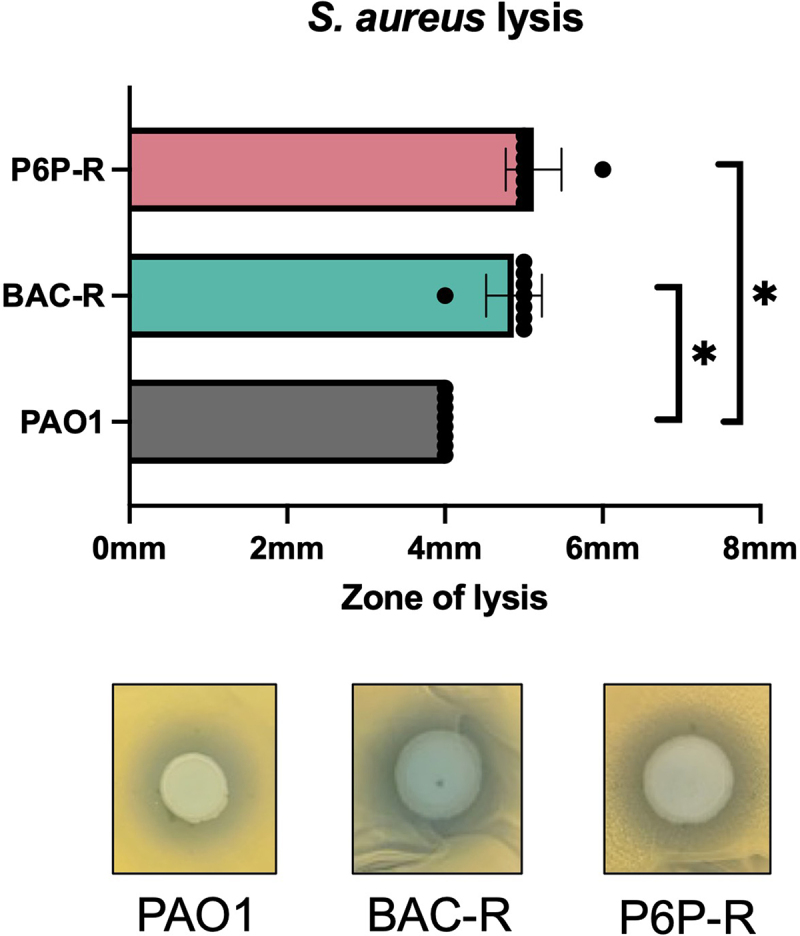
Lysis of *S. aureus* by *P. aeruginosa* PAO1 and cb-resistant strains. Lysis zones generated on *S. aureus* bacterial lawns by cb-resistant strains were measured and compared to the zones generated by parental isogenic strain PAO1. Top panel shows measurements of lysis zones (6 replicates analyzed/strain). Bottom panels show lysis zones on *S. aureus* produced by *P. aeruginosa* cb-resistant and PAO1 parental strains. ****p < 0.05 in two-tailed Student’s t-test compared to parental strain.

We sought to study the effect of BAC and P6P–10,10 resistance on the inter-species relationship of *Staphylococcus aureus* and *P. aeruginosa*. Frequently isolated together, *P. aeruginosa* and *S. aureus* are important pathogens in human disease from wound infections to chronic lung infections [45]. Co-infections have been shown to be more virulent than single species infections [46,47]. To our knowledge, no studies have been performed to assess the effect of disinfectant resistance on interbacterial competition. To interrogate the resistant *P. aeruginosa* phenotype, we conducted lysis experiments to determine whether disinfectant resistance affected the ability of PAO1 to induce lysis in *S. aureus* (Figure 4). In plate assays, BAC- and P6P–10,10-resistant PAO1 strains had greater lysis zones than the parent PAO1 strain. In agreement with our previous results, the BAC-R PAO1 strain produced more bluish-green pigment than the two other PAO1 strains, correlating with the observed increase in pyocyanin production. This secreted factor production may be responsible for the increased lysis. However, despite a less substantial increase in PYO and an observed decrease in PVD, the P6P-R strain had the same lysis effects on *S. aureus*, as measured by zone of lysis. This indicates that there are other secreted virulence factors that may be primarily responsible for causing the zone of lysis in PAO1. Nevertheless, these data indicate that CB resistance can affect important interbacterial relationships, which may have implications for human infections.

### CB-resistant mutants show competitive disadvantage compared to the PAO1 parental strain

Bacterial competition is an important interaction that occurs between and within species and can determine which microorganisms will thrive within a niche. In *P. aeruginosa*, mutant isolates, known as “social cheaters” take advantage of secreted factors by surrounding bacteria without the need to spend resources to produce them themselves, allowing them to thrive. Similar mutants have been isolated in CF patients, highlighting the potential implications for health.

**Figure 5.**
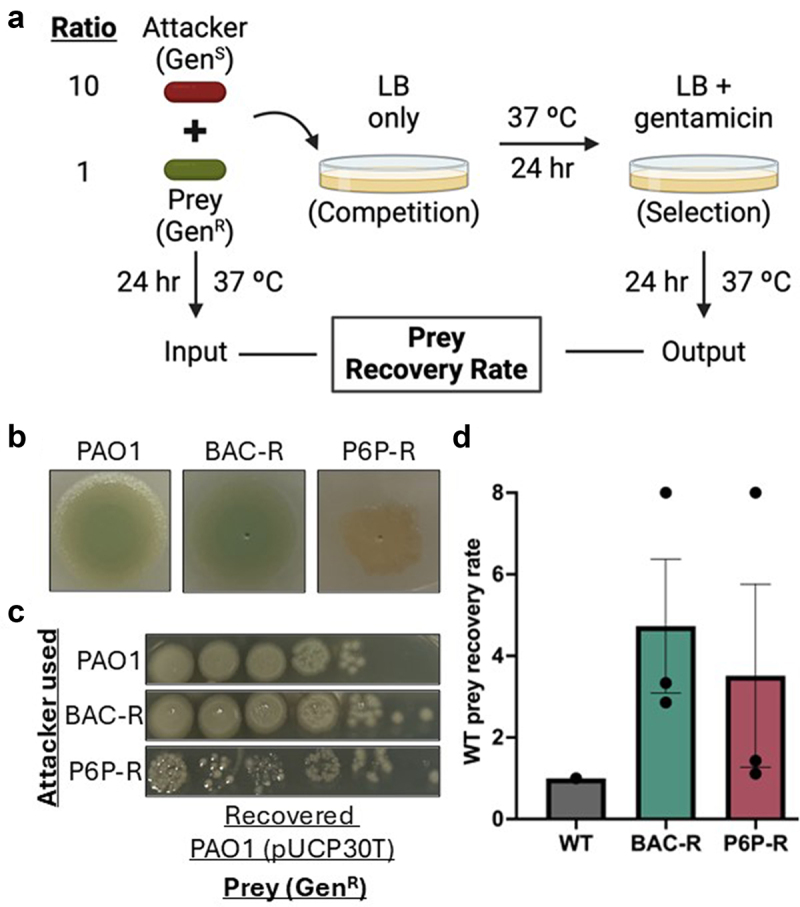
Bacterial competition assays of cb-resistant and PAO1 parental strain. a) schematic of the prey recovery rate interbacterial competition assay. b) different morphologies observed in competition spots between attacker strains PAO1 (WT control), bac-R and P6P-R strains versus PAO1 harbouring a vector conferring gentamicin resistance (prey strain; Gen^R^) on LB agar media after 24 hours using an attacker to prey ratio of 10:1. c) spot plating for recovery of prey strain in LB gentamicin (60 µg/mL) plate for prey recovery rate determination after competition. d) prey recovery rate of competition assays between *P. aeruginosa* PAO1, bac-resistant and P6P-resistant strains against *P. aeruginosa* PAO1 Gen^R^. The mean of three replicates is shown. No statistical significance was observed by two-tailed Student’s *t* test.

Since we observed variations in fitness while growing the CB-resistant strains in different media (Supplemental Figures S2 and S3), we wanted to determine the competitive fitness of these isogenic mutants. To investigate this, we performed a prey recovery rate competition assay [48,49]. This assay consists of co-incubating the strains of interest (“attackers”) with a reporter strain (“prey”) at a fixed ratio and using a selectable marker on the prey strain to select only this strain after competition (Figure 5a). In our case, the prey strain (PAO1 pUCP30T) is gentamicin-resistant, while the attacker strains (PAO1, BAC-R, and P6P-R) are gentamicin-sensitive. The PAO1 wildtype strain without the vector was included as a control. After selection, the prey recovery rate is calculated by comparing the recovered prey in selective media (output) with the prey amount at the beginning of the experiment (input). This competitive assay provides additional information on the fitness of these isogenic strains under mixed culture conditions.

We observed phenotypic differences during our competitive incubation period, especially with our P6P-resistant strain (Figure 5b). While we observed differences suggestive of a competitive advantage of the P6P-resistant strain, the overall prey recovery rate indicated no significant difference compared to the WT control (Figure 5c). The BAC-resistant strain showed an overall lower fitness compared to the WT, as indicated by a higher prey recovery rate compared to the one obtained in the WT control (Figure 5d). These data suggest that resistance to CBs affects competitive fitness within these isogenic strains.

### Evaluation of P. aeruginosa virulence in G. mellonella larvae infection model

**Figure 6.**
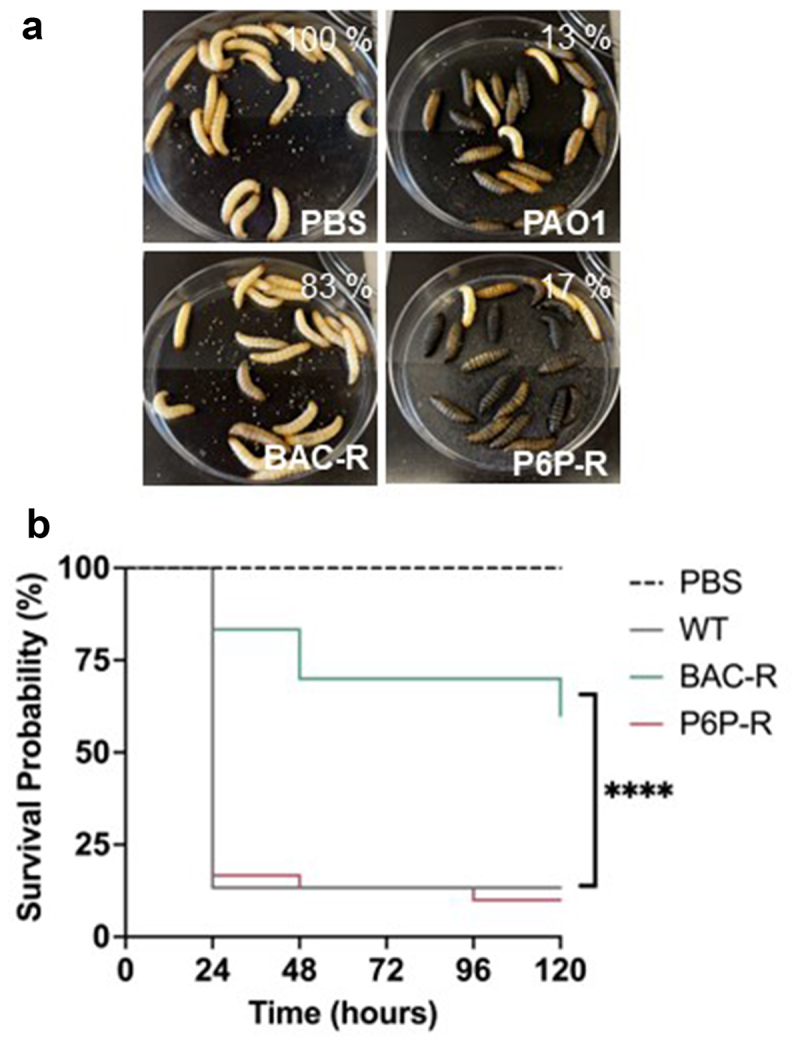
*Galleria. mellonella* larvae infected with cb-resistant and parental PAO1 strains. A) morbidity of *G. mellonella* larvae 24 hours post-infection. Survival (%) at 24 hours post-infection is shown in the top right corner of each picture. B) Kaplan-Meier survival curves of *G. mellonella* larvae monitored over a 5-day period. Two independent experiments were performed with a total of 120 larvae with pbs-injected larvae used as mock infection control (*n* = 30/group). Survival curves were compared using log-rank (mantel-cox) test. ****p < 0.0001.

To evaluate how the production of virulence factors in CB-resistant strains affects virulence *in vivo*, we utilized the *Galleria mellonella* larvae infection model. This system is used to study virulence factor production and virulence in *P. aeruginosa* [50]. As previously reported, we observed that most larvae infected with the parental strain PAO1 died within 24 h when infected with an average dose of 5 CFU/larva, with only 13% of the infected larvae surviving the infection [51]. The larvae infected with the P6P-resistant strain displayed a similar survival probability with 17% of the infected larvae surviving the infection after 24 h (Figure 6a). Interestingly, the larvae infected with the BAC-resistant strain showed a significant decrease in morbidity, with over half of the population surviving infection (60%) throughout the experiment (Figure 6b). This result is particularly interesting since the BAC-resistant strain consistently shows the highest production of different virulence factors. This suggests that the fitness cost imposed by BAC resistance development decreases virulence *in vivo*. In addition, these data indicate that CB resistance development in *P. aeruginosa* differentially affects virulence in this infection model.

## Conclusion

Disinfectants are an important part of our arsenal against infections as they represent the first lines of defense against the spread of pathogens. Cationic biocides stand out as commonly used disinfectants in both household and hospital settings. In this work, we investigated the effect of CB resistance development of the widely used BAC and our next-generation disinfectant P6P–10,10 on *P. aeruginosa* defensive strategies used by the bacteria to protect themselves from antimicrobials and offensive strategies used to thrive against other organisms. While the changes in virulence associated with P6P mutants were modest overall, we found that resistance development to the commonly used disinfectant BAC led to increased production of virulence-associated pigments and pellicle formation, but at a fitness cost. During infection, the BAC-resistant mutant showed decreased virulence, likely due to the fitness burden imposed by resistance. Nevertheless, caution should be taken when interpreting these results since compensatory mutations could remove the fitness cost imposed and allow these resistant mutants to thrive. Even though several virulence factors are increased in some of these CB-resistant strains *in vitro*, the regulation and production of these factors *in vivo* warrants further investigation. Understanding how exposure to disinfectants affects virulence in highly pathogenic bacteria such as *P. aeruginosa* is crucial for the establishment of effective disinfection protocols that avoid promoting the development of potentially more virulent strains.

## Materials and methods

### Bacterial strains and growth conditions

*P. aeruginosa* strains were streaked onto lysogeny broth (LB) agar (Sigma-Aldrich 1,102,830,500) plates and incubated (NuAire, Plymouth, MN) at 37 ºC overnight. Single colonies were used to inoculate liquid cultures and incubated for 18–24 h at 37 ºC with shaking. *P. aeruginosa* clinical isolates were obtained from the Multidrug-Resistant Organism Repository and Surveillance Network (MRSN).

Growth curves were performed in 96-well flat-bottom plates (Falcon®, 351172) with shaking. OD_600_ was measured every 10 min and growth was monitored over 24 h. Growth curve experiments were performed on different days with independent biological replicates with at least six technical replicates per strain/condition. MOPS and M63 minimal media were prepared as previously described and supplemented with glucose as a carbon source [52]. Synthetic cystic fibrosis medium (SCFM) was prepared as previously described with the addition of N-acetyl glucosamine [53,54].

### Statistical criteria

The experimental data were analyzed using the GraphPad Prism 9.0 software (San Diego, CA). When the value of *p* < 0.05, it was considered statistically significant.

### Resistance selection

Disinfectant-resistant mutants were generated via serial passing of *P. aeruginosa* PAO1, MRSN6220, MRSN6241, MRSN409937, and MRSN5524 under increasing concentrations of either BAC or P6P–10,10 as previously reported [55]. First, the overnight culture of PAO1 in Difco™ Mueller-Hinton Broth (Sigma Aldrich, DF0757-17-6) was diluted to a concentration of 106 CFU/mL, according to the OD_600_ value. Six 100 μL concentrations of the compound, ranging from four-fold the MIC to half the MIC, were then inoculated with 100 μL of the dilute culture and incubated for 24 h at 37 ºC. After 24 h, a 2.0 μL aliquot from the highest concentration displaying growth was diluted 1:100 in fresh MHB (Sigma-Aldrich, DF0757-17-6) and fresh sample of compounds. This process was repeated for 14 total serial passages. Experiments were performed on biological triplicates.

### Cross resistance MIC assay

To determine the MIC values, antibiotics were serially diluted two-folds from stock solutions (1.0 mM) to yield 12 100 μL test concentrations. *Pseudomonas aeruginosa* strains were streaked onto lysogeny (LB) agar (Sigma-Aldrich 1,102,830,500) plates and incubated (NuAire, Plymouth, MN) for 18 h at 37°C. Single colonies were used to inoculate 5 mL of Falcon® Mueller–Hinton (MHB) broth (Sigma-Aldrich, DF0757-17-6), and cultures were grown at 37°C for 18 h with shaking. Overnight cultures were diluted 1:100 in MHB and regrown to a mid-exponential phase as determined by the optical density at 600 nm (OD_600_). All cultures were diluted to ca. 10^4^ CFU/mL in MHB, and 100 μL were inoculated into each well of a U-bottom 96-well plate (Avantor, 734–2782) containing 100 μL of antibiotic solution. Plates were incubated at 37°C for 24 h upon which wells were evaluated visually for bacterial growth. The MIC was determined to have the lowest concentration of compounds resulting in no bacterial growth visible to the naked eye based on the mean in three independent experiments. MHB media and aqueous DMSO (Millipore Sigma, MX1458–6) controls were conducted for each strain. Antibiotics tested were amikacin disulfate (Alfar Aesar, J63862.14), gentamycin sulfate (Millipore Sigma, G1264-250 MG), tobramycin (Millipore Sigma, PHR1079), aztreonam (TCI Chemicals, A2466), ceftazidime (Combi-Blocks, QV-7534), cefepime HCl (Chem-Impex 15,144), imipenem monohydrate (Combi-Blocks, QC-2985), meropenem trihydrate (Combi-Blocks, QH-8889), ciprofloxacin (Enzo Life Sciences, ALX-380-287-G025), levofloxacin (Alfa Aesar, J66943.06), colistin sulfate (Millipore Sigma, C4461-100 MG), and polymyxin B sulfate (Oakwood Chemical, QC-8583). All antibiotics were dissolved in a 1:10 dilution of DMSO:water to create 1.0 mM solutions.

### Alginate quantification

Alginate quantification assay was performed following the procedure described by Chotirmall *et al*. with minor modifications [56,57]. *Pseudomonas aeruginosa* strains were streaked onto lysogeny (LB) agar (Sigma-Aldrich 1,102,830,500) plates and incubated (NuAire, Plymouth, MN) for 18 h at 37°C. Single colonies were used to inoculate 5 mL of Miller LB broth (VWR, TS61187–5000), and cultures were grown at 37°C for 18 h with shaking. 1 M NaCl (Sigma-Aldrich, SX0420–5) was added to the overnight culture in a 1:1 ratio and vortexed. The cultures were centrifuged (Eppendorf, Enfield, CT) at 10 000 rpm for 30 min. 2% cetylpyridinium chloride (Sigma-Aldrich, C0732-100 G) was added to the supernatant in a 2:1 ratio to allow for alginate precipitation. For the collection of alginate, the mixture was centrifuged at 10 000 rpm for 10 minutes at room temperature and resuspended in 500 μL of −20°C isopropanol (Fisher Scientific, A426P–4) for 1 h. The mixture was centrifuged at 10 000 rpm at 4°C for 10 min. The remaining isopropanol was washed by dissolving the alginate pellet in water and lyophilized (Labconco, Kansas City, MO). The alginate pellet was resuspended in 500 μL of 1 M NaCl and heated to 60°C. 50 μL of the alginate solution was added to 200 μL of 25 mM boric acid (Sigma-Aldrich, B0394-500 G)/sulfuric acid (EMD Millipore 258,105-500 ML) (2 M H_3_BO_3_ in sulfuric acid), and the mixture was heated to 100°C for 10 min. The mixture was cooled for 15 min, and 50 μL of 0.125% carbazole (Sigma-Aldrich, C5132-100 G) in 100% ethanol (Decon Labs, 2705SG) was added. The solution was reheated to 100°C for 10 min. Once cooled, the quantification of alginate was determined spectrophotometrically at 550 nm using a BioTek Synergy H1 Hybrid plate reader (Santa Clara, CA).

### Biofilm quantification assay

*Pseudomonas aeruginosa* strains were streaked onto lysogeny (LB) agar (Sigma-Aldrich 1,102,830,500) plates and incubated (NuAire, Plymouth, MN) for 18 h at 37°C. Single colonies were used to inoculate 5 mL of Miller LB broth (VWR, TS61187–5000), and cultures were grown at 37°C for 18 h with shaking. Overnight cultures were diluted 1:100 in M63 minimal media supplemented with 2% (w/v) glucose (Sigma-Aldrich, G8270-100 G) [58]. Diluted cultures were added to surface-treated 96-well flat-bottom microtiter plates (Corning Incorporated, 3598). Plates were incubated at 37°C for 24 h at which time the cell media was aspirated off. Wells were washed twice with 200 μL of phosphate buffer solution (PBS) and dried for 10 min. The wells were incubated for 15 min with 200 μL of 0.1% (w/v) crystal violet (VWR, 0528-500 G) in DI H_2_O. Excess crystal violet was removed by aspirating off the liquid, and the wells were rinsed twice with 200 μL of PBS. Crystal violet stained biofilms were solubilized with 200 μL of 70% (w/v) ethanol (Decon Labs, 2705SG) in DI H_2_O and allowed to incubate at room temperature for 10 min to allow for full dissolution. Then, 100 μL was transferred to a fresh flat-bottom 96-well plate (Falcon®, 351172) for absorbance measurement at 570 nm using a BioTek Synergy H1 Hybrid plate reader (Santa Clara, CA). Biological triplicates were performed with 12 technical replicates using media control.

### Pellicle quantification assays

*Pseudomonas aeruginosa* strains were streaked onto lysogeny (LB) agar (Sigma-Aldrich 1,102,830,500) plates and incubated (NuAire, Plymouth, MN) for 18 h at 37°C. Single colonies were used to inoculate 5 mL of Miller LB broth (VWR, TS61187–5000), and cultures were grown at 37°C for 18 h with shaking. Overnight cultures were diluted by 1:100 in LBs and transferred onto a non-surface treated 96-well plate (Falcon®, 351172) with pegged lid (Nunc^TM^, 445497). Plates were incubated at 37°C for 24 h inside a secondary container to prevent undesired evaporation and processed as previously described [35]. To remove the planktonic cells, the lid was carefully removed and placed into a new 96-well plate with 300 µL of PBS for 30–60 s prior to staining with 300 µL of 0.1% (w/v) crystal violet for 5 min in a different plate. After staining, excess crystal violet was removed by placing the lid in a 96-well plate with 300 µL of PBS in a new plate, and then dried for 10 min with pegs facing upward inside the biosafety cabinet. Finally, the pegged lid was distained in a 96-well plate containing 300 µL of 30% (v/v) acetic acid for 5 min. 200 µL were transferred to a new plate, and the absorbance was measured at a wavelength of 570 nm. Data were corrected with blank values, and the average values of 3 biological replicates were analyzed.

### Motility assay

Motility assays were performed following the procedure described by Cullen *et al*. with minor modifications [59]. *Pseudomonas aeruginosa* strains were streaked onto lysogeny (LB) agar (Sigma-Aldrich 1,102,830,500) plates and incubated (NuAire, Plymouth, MN) for 18 h at 37°C. Single colonies were used to inoculate 5 mL of Miller LB broth (VWR, TS61187–5000), and cultures were grown at 37°C for 18 h with shaking. Swimming motility was assessed by inoculating the surface of LB media (Sigma-Aldrich 1,102,830,500) petri plate supplemented with 0.3% (w/v) Bacto™ agar (Fisher Scientific, DF0479-17-3) with overnight culture using a sterile 10 μL pipette tip. Swimming plates were incubated at 37°C for 18 h. Swarming motility was assessed by inoculating the surface of LB media (Sigma-Aldrich 1,102,830,500) supplemented with 0.5% (w/v) Bacto™ agar (Fisher Scientific, DF0479-17-3) plate with overnight culture using a sterile 10 μL pipette tip. Swarming plates were incubated at 30°C for 18 h. Diameters of swimming and swarming zones were calculated by averaging two perpendicular measurements. Two biological replicates per strain were performed.

### S. aureus Lysis

Adapted from Mashburn *et al*., lysis of *S. aureus* on petri plates (VWR 25,384–342) was performed by swabbing a lysogeny (LB) plate (Sigma-Aldrich 1,102,830,500) with an overnight culture of *S. aureus* [60]. After drying, 5 μl of an overnight culture of *P. aeruginosa* was spotted onto the petri plate, dried, and incubated at 37°C for 24 h. Zones of lysis were subsequently measured and imaged.

### Pyoverdine quantification

Pyoverdine (PVD) quantification was performed following the procedure described by Hoegy *et al*. with minor modifications [61]. Prior to completing the quantification, the parameters for the plate reader (BioTek Synergy H1 hybrid, Santa Clara, CA) were established as excitation wavelength at 400 nm and emission wavelength at 447 nm. The read height was 7.00 mm, and the temperature was set at 37°C. Each fluorescence measurement occurred with the lid on the plate. *Pseudomonas aeruginosa* strains were streaked onto lysogeny (LB) agar (Sigma-Aldrich 1,102,830,500) plates and incubated (NuAire, Plymouth, MN) for 18 h at 37°C. Single colonies were used to inoculate 5 mL of Difco™ Mueller–Hinton (MHB) broth (Sigma-Aldrich, DF0757-17-6), and cultures were grown at 37°C for 18 h with shaking. 100 μL of overnight culture was added to an Eppendorf tube (Fisher Scientific, 02-681-320) followed by 900 μL of Tris-HCl pH 8.0 buffer (Fisher Scientific, BP1758–100), and the solution was mixed. 100 μL of the mixture was inoculated into the well of a black flat bottom 96-well plate (Nunclon 137,101) for fluorescence measurement of apo PVD. When excited at 400 nm, the fluorescence emission of apo PVD is 447 nm; PVD in complexes with iron will not be fluorescent.

### Prey competitive fitness experiments

Inter-bacterial competition assays were performed as previously described with modifications [48,49]. *P. aeruginosa* PAO1 pUCP30T (Gen^R^) served as prey and readout of the experiment. Isogenic BAC-R and P6P-R strains served as attacker strains with PAO1 without the vector conferring resistance to gentamicin used as a control. A 10:1 attacker:prey ratio was used for these studies. Overnight cultures of bacterial strains were diluted 100-fold and grown to an OD_600_ of 1. Cultures were normalized to an OD_600_ of 0.4 right before the start of the experiment. The initial colony forming units (CFUs) counts were determined and used as input. Our *P. aeruginosa* pUCP30T (Gen^R^) prey strain was selected in Miller LB broth (VWR, TS61187–5000) supplemented with gentamicin (Millipore Sigma, G1264-250 MG) 60 µg/mL after competition and used as output. The prey recovery rate was calculated by dividing the CFUs counts of the output by the CFUs of the input.

### Pyocyanin quantification

Adapted from Taylor *et al*., overnight cultures of *P. aeruginosa* were grown from single colonies in Miller lysogeny (LB) medium (VWR, TS61187–5000) and grown with shaking at 37°C for 20 h [62]. 1 mL of each culture was subjected to centrifugation (Eppendorf, Enfield, CT) at 13 000 rpm for 3 min. The clarified supernatants were collected, and the OD_695_ was measured on a BioTek Synergy H1 Hybrid plate reader (Santa Clara, CA). The pellets were resuspended in PBS, and the OD_600_ was measured to determine the cell density of each sample. Pyocyanin production was determined by normalizing OD_695_ of the clarified supernatant to the OD_600_ of the resuspended pellet.

### Galleria mellonella infections

Healthy *G. mellonella* 5^th^ instar larvae (SpeedyWorm) of similar size (180−250 mg) were carefully selected for infection, and larvae displaying signs of disease (e.g. black spots) were discarded [51]. Larvae were infected with an average of 5 CFU per worm (10 µL) using 30-gauge needles (BD 305,106) and 25-µL syringe (Hamilton 80,401). Infections were monitored over a 5-day period for signs of morbidity (melanization, akinesia, inability to right itself). A total of 30 larvae were infected per group, including a PBS mock-infected control group. Two independent experiments were performed with two different batches of *G. mellonella* larvae.

## Supplementary Material

Supplemental Material

## Data Availability

The authors confirm that the data supporting the findings of this study are available within the article, its supplementary materials, and through Mendeley Data (https://doi.org/10.17632/rhfwf2rycs.2). MRSN strains are available directly from Dr Patrick McGann.
