# Supplementary material for: Adaptations to cationic biocide exposure differentially influence virulence factors and pathogenicity in Pseudomonas aeruginosa
Source: Virulence. 2024 Sep 16;15(1):2397503. doi: 10.1080/21505594.2024.2397503 (PMC11407422; doi:10.1080/21505594.2024.2397503)
Supplement: Supplemental Material [file KVIR_A_2397503_SM9736.pdf]

Adaptations to cationic biocides exposure differentially influence virulence factors and pathogenicity in *Pseudomonas aeruginosa*

Germán G. Vargas-Cuebas[a†], Christian A. Sanchez[b†], Elise L. Bezold[b], Gabrielle M. Walker[b], Shehreen Siddiqui[b], Kevin P.C. Minbiole[c], and William M. Wuest[b]*

[a]*Department of Microbiology and Immunology, Emory University School of Medicine, 1510 Clifton Rd, Atlanta, GA USA;*

[b]*Department of Chemistry, Emory University, 1515 Dickey Dr. Atlanta, GA USA;*

[c]*Department of Chemistry, Villanova University, 800 E. Lancaster Ave. Villanova, PA USA;*

* *Corresponding author email: wwuest@emory.edu; kevin.minbiole@villanova.edu*

[†] *denotes equal contribution*

Supplementary Figures

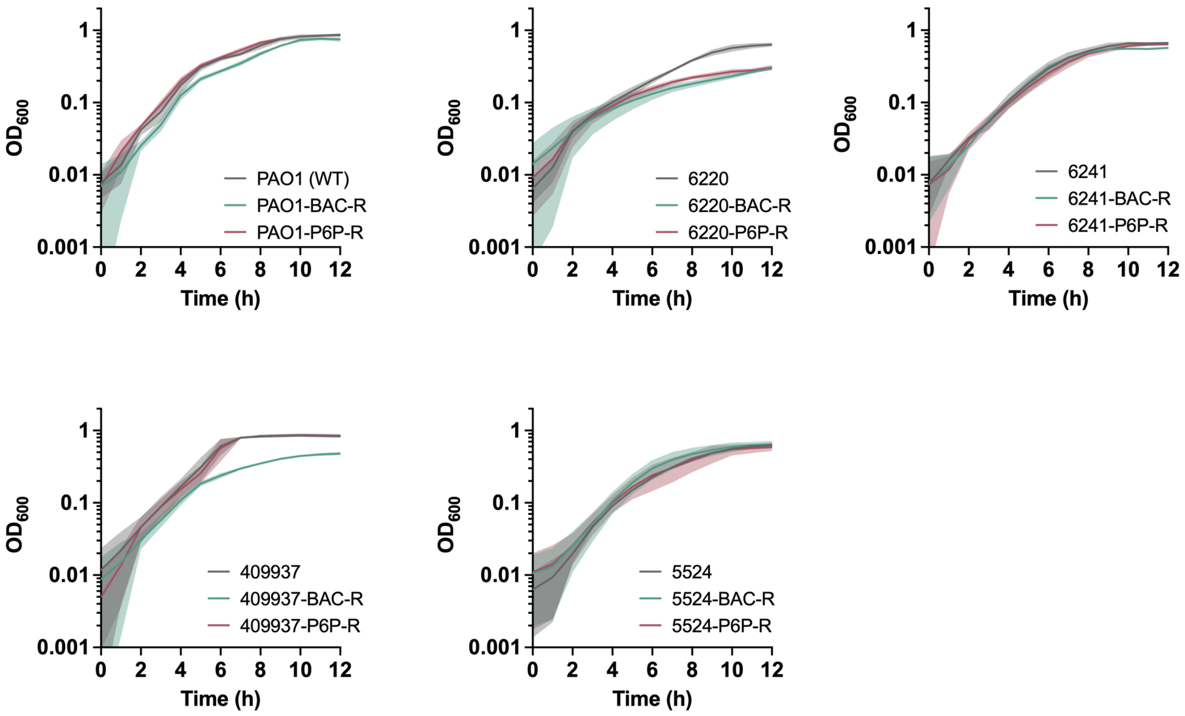

Figure S1: Growth curves performed in lysogeny broth (LB) with CB-resistant and parental strains. CB-resistant and parental strains growth was monitored over a 36-hour period in 96-well plates with shaking at 37 ºC, with OD_{600} measurements every 10 minutes. Only growth for 12 hours is shown to facilitate comparisons between strains. Each plot shows mean values of OD_{600} for the wild-type (WT) parental strain (gray), and the derived BAC-resistant (teal) and P6P-resistant (dark coral) strains. The mean value (solid lines) and standard deviation (shaded area) of 3 biological replicates for each strain is shown.

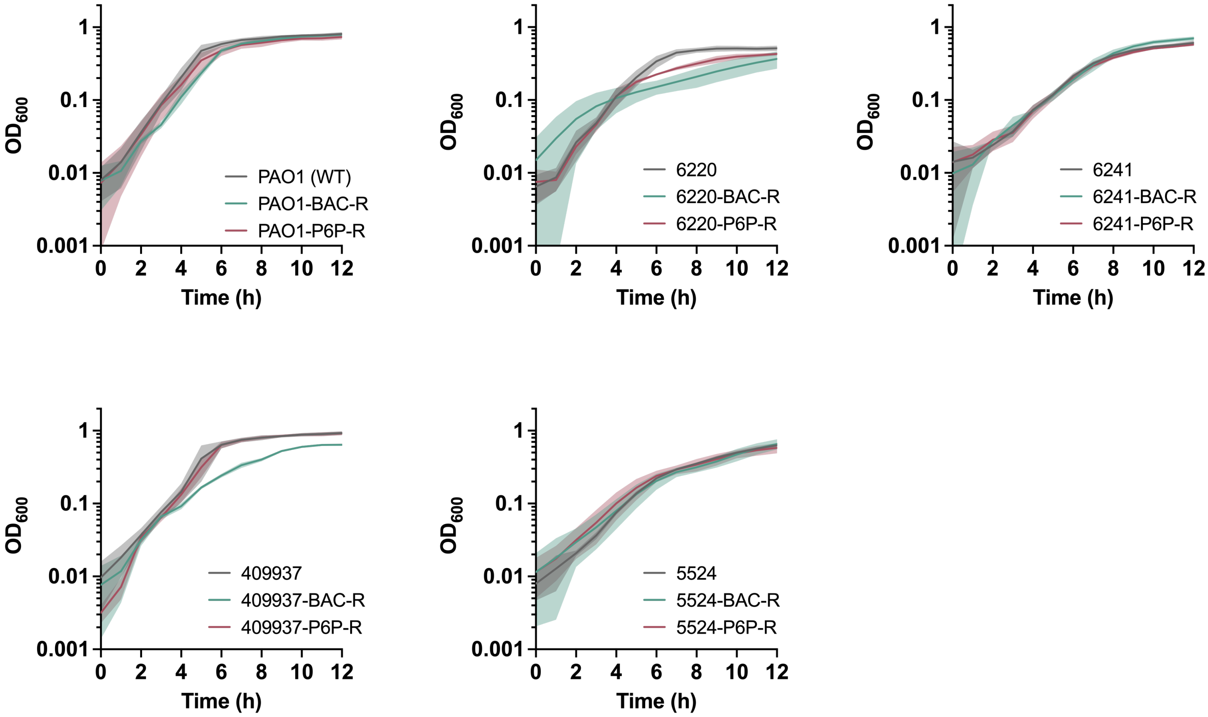

Figure S2: Growth curves performed in synthetic cystic fibrosis media (SCFM) with modifications with CB-resistant and parental strains.[1,2] CB-resistant and parental strains growth was monitored over a 36-hour period in 96-well plates with shaking at 37 ºC, with OD_{600} measurements every 10 minutes. Only growth for 12 hours is shown to facilitate comparisons between strains. Each plot shows mean values of OD_{600} for the wild-type (WT) parental strain (gray), and the derived BAC-resistant (teal) and P6P-resistant (dark coral) strains. The mean value (solid lines) and standard deviation (shaded area) of 3 biological replicates for each strain is shown.

Cross Resistance MICs, μM												
Strain	Aminoglycosides			Monobactam	Cephalosporins		Carbapenems		Fluoroquinolones		Polymyxins	
	AMK	GEN	TOB	ATM	CAZ	FEP	IPM	MEM	CIP	LVX	CST	PMB
**PAO1**	4	2	0.5	16	2	1	2	1	2	8	4	0.25
PAO1 BAC^R	8	8	2	8	2	1	2	2	1	4	2	0.25
PAO1 P6P^R	4	4	1	8	4	1	8	2	4	8	2	0.25
**6220**	250	125	>250	>250	>250	>250	>250	>250	125	250	4	0.25
6220 BAC^R	250	125	>250	125	>250	125	>250	250	63	125	2	0.25
6220 P6P^R	250	125	>250	>250	>250	>250	>250	>250	125	125	4	0.25
**6241**	8	125	63	>250	>250	250	32	32	125	125	2	0.25
6241 BAC^R	1	16	8	>250	>250	250	32	32	8	16	4	0.25
6241 P6P^R	8	125	125	>250	>250	250	32	32	125	125	2	0.25
**409937**	16	8	4	>250	>250	250	8	32	16	63	4	0.25
409937 BAC^R	4	2	1	250	125	63	2	4	8	63	2	0.25
409937 P6P^R	1	0.25	0.25	>250	>250	125	16	16	16	63	2	0.25
**5524**	16	>250	250	125	16	32	16	63	4	32	2	0.25
5524 BAC^R	16	>250	125	125	16	63	16	32	2	16	4	0.5
5524 P6P^R	4	250	32	125	16	32	16	32	2	16	2	0.25

Antibiotic abbreviations: amikacin (AMK), gentamycin (GEN), tobramycin (TOB), aztreonam (ATM), ceftazidime (CAZ), cefepime (FEB), imipenem (IPM), meropenem (MEM), ciprofloxacin (CIP), levofloxacin (LVX), colistin sulfate (CST), polymyxin B (PMB)

Figure S3: Full minimum inhibitory concentrations (MIC) of parental (bold) and CB-resistant (R) strains of *P. aeruginosa*.

References

[1] Meirelles LA, Perry EK, Bergkessel M, et al. Bacterial defenses against a natural antibiotic promote collateral resilience to clinical antibiotics. PLoS Biol. 2021 Mar;19(3):e3001093.

[2] Palmer KL, Aye LM, Whiteley M. Nutritional cues control Pseudomonas aeruginosa multicellular behavior in cystic fibrosis sputum. J Bacteriol. 2007 Nov;189(22):8079-87.
